# Dispatch guideline adherence and response interval—a study of emergency medical calls in Norway

**DOI:** 10.1186/s12873-016-0105-2

**Published:** 2016-10-13

**Authors:** Eirin N. Ellensen, Torben Wisborg, Steinar Hunskaar, Erik Zakariassen

**Affiliations:** 1 Department of Research, Norwegian Air Ambulance Foundation, PB 94, 1441 Drøbak, Norway; 2 Department of Global Public Health and Primary Care, University of Bergen, PB 7800, 5020 Bergen, Norway; 3 Anaesthesia and Critical Care Research Group, Faculty of Health Sciences, University of Tromsø, Tromsø, 9037 Norway; 4 Norwegian National Advisory Unit on Trauma, Oslo University Hospital, Oslo, 0450 Norway; 5 Department of Anesthesia and Intensive Care, Hammerfest Hospital, Finnmark Hospital Trust, Hammerfest, 9613 Norway; 6 National Centre for Emergency Primary Health Care, Uni Research Health, Kalfarveien 31, Bergen, 5018 Norway

**Keywords:** Telephone recordings, Guideline adherence, Dispatch, Emergency medicine

## Abstract

**Background:**

The Emergency Medical Communication Centre (EMCC) operators in Norway report using the Norwegian Index for Medical Emergency Assistance (Index), a criteria-based dispatch guideline, in about 75 % of medical emergency calls. The main purpose of a dispatch guideline is to assist the operator in securing a correct response as quickly as possible. The effect of using the guideline on EMCC response interval is as yet unknown. We wanted to ascertain an objective measure of guideline adherence, and explore a possible effect on emergency medical dispatch (EMD) response interval.

**Methods:**

Observational cross-sectional study based on digital telephone recordings and EMCC records; 299 random calls ending in acute and urgent responses from seven strategically selected EMCCs were included. Ability to confirm location and patient consciousness within an acceptable time interval and structural use of criteria cards were indicators used to create an overall guideline adherence variable. We then explored the relationship between different levels of guideline adherence and EMD response interval.

**Results:**

The overall guideline adherence was 80 %. Location and patient consciousness were confirmed within 1 min in 83 % of the calls. The criteria cards were used systematically as intended in 64 % of the cases. Total median response interval was 2:28, with 2:01 for acute calls and 4:10 for urgent calls (*p* < 0.0005). Lower guideline adherence was associated with higher EMD response interval (*p* < 0.0005).

**Conclusion:**

The measured guideline adherence was higher than previously reported by the operators themselves. Patient consciousness was rapidly confirmed in the majority of cases. Failure to use Index criteria as intended result in delayed ambulance dispatch and a potential risk of undertriage.

**Electronic supplementary material:**

The online version of this article (doi:10.1186/s12873-016-0105-2) contains supplementary material, which is available to authorized users.

## Background

The Emergency Medical Communication Centres (EMCCs) are important links in the chain of prehospital emergency medicine in Norway. They are responsible for receiving and triaging phone calls made to the toll-free public emergency medical telephone number 113, dispatching and administering the entire ambulance fleet including the air ambulance service, alerting primary care doctors on-call, and facilitating the communication between prehospital and in-hospital health personnel in acute medical situations. The EMCCs are manned by registered nurses who answer the 113 calls, and ambulance personnel who coordinate the ambulance fleet. Norwegian Index for Medical Emergency Assistance (Index) is a criteria-based dispatch (CBD) guideline that supplements the medical knowledge and experience of the individual telephone operator during the dispatch process [1]. The criteria based approach to medical dispatch allows for a more open and dynamic interaction between caller and operator than stricter algorithm-based protocols like the Medical Priority Dispatch System (MPDS). CBDs have been criticized because it is difficult to monitor the dispatch process and determine guideline adherence, thereby complicating both quality improvement processes and research [2–4].

A 2013 systematic review found that there is an lack of studies on an international basis addressing the two principal systems concerning guideline adherence and protocol compliance [5]. A 2014 study compared cardiac arrest calls processed by a MPDS protocol in Richmond, USA, and the Norwegian CBD guidelines [6]. Indicators for protocol compliance/guidelines adherence were clarification of consciousness (100 and 97 % for MPDS and CBD, respectively), and detection of respiratory arrest (100 and 98 %, respectively). The generalization of these results to the protocol and guidelines as a whole is limited, given the narrow patient groups on which the study focused. A national questionnaire survey among the Norwegian EMCC operators showed a wide range in self-reported use of the Index, not only on the level of individual operators, but also on the EMCC level [2]. The mean self-reported use of the Index found in that study corresponded to operators’ use of Index in approximately 75 % of all medical emergency phone calls. Another Norwegian study from 2008 focused on impaired consciousness and intoxication [7]. The study revealed a discrepancy between the use of the Index obtained through operators’ questionnaires and recorded phone calls. Some 99 % of the operators claimed to use the Index, while the recordings showed that the level of consciousness was clarified in only 64 % of the contacts.

The aim of this study was to measure guideline adherence objectively through evaluation of real telephone recordings. Secondly, we wanted to investigate a possible association between use of the Index and the emergency medical dispatch (EMD) response interval.

## Method

### The Norwegian Index for Medical Emergency Assistance (Index)

The Index presupposes medical knowledge and is to be used by healthcare staff who are educated and experienced. It consists of a Start page and 36 symptom-based cards. The operator begins at the Start page to obtain important information on location, the state of consciousness and the situation at hand before deciding on immediate acute ambulance dispatch, life-saving instructions, or if the time allows; the most suitable symptom card to continue exploring the situation. The first three cards (Unconscious, Unconscious child and Obstruction of airways) are mainly instructional, with a focus on cardiopulmonary resuscitation (CPR). Each of the remaining cards consists of a list of criteria, corresponding responses, additional questions, caller advices, and supporting instructions for health personnel at scene. The guidelines use three different colour categories according to urgency: red, yellow and green. The colour red is for acute and life-threatening situations, yellow is for urgent and potentially life-threatening situations, and green is for non-urgent situations. The list of criteria decline in urgency from the top down, and the operators are instructed to start from the top at the most acute criterion, reformulate into questions and work their way down the page until one criterion is finally met. As each criterion is predefined as acute, urgent or non-urgent, confirmation of a criterion automatically releases a correlated response.

### Study design and study sample

We performed an observational cross-sectional study of acute and urgent 113 calls during a 72-h period in late August 2011. Nine of Norway’s 19 EMCCs were invited to participate; one did not have recordings from the inclusion period, one did not respond and seven agreed to join the study. The selection was strategically based on diversity in population density (5–210 inhabitants/km^2^), contact rates (36–75 contacts/1 000 inhabitants/year), geographic area (all regions represented) and self-reported use of Index at EMCC level (3.7-4.4). “Self-reported use of Index” originated from a previously published questionnaire study [2]. The EMCC operators were given a Likert scale of 11 questions on how often they used different parts of Index, with 5 response formats (1 = never, 2 = seldom, <25 %, 3 = sometimes, 4 = often, >75 %, 5 = always), giving each operator and EMCC a mean value on use of Index between 1 (=never) and 5 (=always) (2). The selected study sample had a contact rate of 57/1 000 inhabitants per year, compared to the national contact rate of 56/1 000 [2]. The mean self-reported use of Index in the sample was 3.86 (SD 0.40), slightly below the national value of 3.95 (SD 0.39) and corresponded to the response format “often, >75 %” (=4) [2].

### Data collection

We collected a random sample of 50 digitalized call recordings from each of the five largest EMCCs, and 20 and 30 from the two smallest EMCCs. Randomization was achieved through a number generator (www.random.org). Time records, set dispatch criteria and urgency were obtained from Acute Medical Information System (AMIS), the software program used to register and document each contact. One call recording was excluded because of missing AMIS information.

We used a pre-developed form when analysing the call recordings [Additional file 1], based on an internal form used for guidelines adherence and quality control by one of the participating EMCCs. Each call recording was analysed on the basis of six different indicators; (1) confirmation of caller phone number, (2) location, (3) patient consciousness, (4) patient responsiveness, (5) criteria compliance, and (6) communication problems. Both information given spontaneously by the caller and information asked specifically for by the operator were registered, but merged for analyses.

Indicators 1–4 were from the Start page. This information was categorized into “within 1 min”, “within 2 min” or “not at all”. Information gained after 2 min was interpreted as negative. Criteria compliance (indicator 5) was measured by the number of criteria above the set criterion not accounted for during the call. Hence a criteria compliance value of 0 means that all criteria above the one set by the operator were accounted for during the call, whereas increasing criteria compliance values mean that the operator has overlooked some more urgent criteria. The variable “Criteria compliance” was grouped in 0, 1 and 2 and more. Indicator 6 (communication problems) was registered as positive if the operator failed to understand what the caller was trying to communicate, as assessed by the researcher.

The main outcome variable “Overall guideline adherence” was composed from the three variables confirmation of location, confirmation of consciousness and criteria compliance. Each variable was dichotomized, and information gained within 1 min for the two first variables, and zero or one unchecked criteria above the set criterion were interpreted as positive. The variables were weighted equally, composing a measured guideline adherence score of 0 (no positive) to 3 (all positive). The secondary outcome variable “EMD response interval” was defined as time from the call was answered until an ambulance was dispatched [8]. Time intervals are reported as minutes:seconds (mm:ss). The data are available as an additional file [Additional file 2].

### Statistical analysis

Continuous data are presented as means (SD) or medians (interquartile range, IQR) for symmetric and skewed data respectively. Rates are calculated as contacts per 1 000 inhabitants per year. Response interval was analysed as a continuous variable (seconds). Q-Q plots were used to check the outcome variables for normal distribution. Spearman rho was used to investigate correlations between the main variable and secondary variables. One-way analysis of variance (ANOVA) was used to compare mean scores on the main outcome variable between EMCC centres, and Mann-Whitney U test and Kruskal-Wallis tests to compare scores on the secondary outcome variable EMD response interval.

We performed statistical analyses using Statistical Package for the Social Sciences (IBM SPSS Statistics 23). *P*-values < 0.05 were considered statistically significant.

## Results

Of the 299 call recordings included in the study, 279 resulted in an ambulance dispatch; 99 % of the 174 acute contacts resulted in an ambulance dispatch (*n* = 173), whereas 87 % of the 125 urgent contacts released an ambulance dispatch (*n* = 106). Table 1 shows an overview of the six Index indicators measured in the study.

**Table 1:** The six Index indicators and their effect on EMD response interval^a^

	All calls (*N* = 299)		Ambulance dispatches (*N* = 279)				
					EMD response interval (min:sec)^a^		
	N	%	N	%	Median	IQR^b^	*P*-value
Caller’s phone number confirmed							0.973
Within 1 min	59	20	56	20	2:49	2:01	
1–2 min	19	6	18	6	2:21	2:31	
After 2 min/none	221	74	205	73	2:28	2:54	
Location confirmed							0.225
Within 1 min	247	83	237	85	2:23	2:36	
1–2 min	23	8	22	8	3:00	2:52	
After 2 min/none	29	10	20	7	3:11	4:27	
Patient’s consciousness							0.429
Within 1 min	249	83	231	83	2:23	2:32	
1–2 min	24	8	23	8	3:12	4:27	
After 2 min/none	26	9	25	9	2:39	3:00	
Patient’s responsiveness							0.086
Within 1 min	240	80	224	80	2:23	2:33	
1–2 min	29	10	26	9	3:57	3:56	
After 2 min/none	30	10	29	10	2:03	2:26	
Criteria compliance^c^							0.001
All criteria confirmed	191	64	184	66	2:17	2:17	
1 criterion unconfirmed	33	11	29	10	2:39	2:04	
2+ criteria unconfirmed	75	25	66	24	3:48	4:59	
Communication							0.691
Good	256	86	241	86	2:33	2:39	
Difficult^d^	43	14	38	14	2:22	2:48	

^a^Emergency Medical Dispatch response interval: Time from 113 call is answered until an ambulance is dispatched

^b^ *IQR* Interquartile range

^c^How many criteria above the one set by the operator is not answered for during the call

^d^Difficult communication includes language difficulties, non cooperative caller and caller not on scene (third hand information)

### Adherence to guideline

The mean overall guideline adherence was 2.41 (0.73), 80 % of maximum value of 3. The EMCCs differed in guideline adherence from 75 to 89 % (Table 2), but one-way ANOVA showed no statistically significant differences between the centres (*p* = 0.073).

**Table 2:** Overall guideline adherence distribution among the EMCCs

	Guideline adherence value distribution (%)				Mean value (SD)	% of maximum
EMCC no	0	1	2	3		
1	3	7	40	50	2.37 (0.77)	79
2	5	0	35	60	2.50 (0.76)	83
3	0	10	46	44	2.34 (0.66)	78
4	2	16	32	50	2.30 (0.81)	77
5	0	22	31	47	2.24 (0.80)	75
6	2	10	28	60	2.46 (0.76)	82
7	0	2	28	70	2.68 (0.51)	89
All	1	11	34	54	2.41 (0.73)	80

There was a low, but statistically significant correlation, between overall guideline adherence and urgency (rho = 0.27, *p* < 0.0005), revealing that acute contacts had higher levels of overall guideline adherence, compared with urgent contacts. There was no statistically significant correlation between overall guideline adherence and self-reported use of the Index, EMCC contact rate or population density.

### EMD response interval

Location, telephone number or consciousness status confirmations within 1, 2 min or not at all did not affect the EMD response interval to any statistically significant extent. Decreasing criteria compliance increased the response interval (*p* = 0.001) (Table 1). Difficulties in communication due to language, non-cooperation or caller not on the scene did not affect EMD response interval.

The set level of urgency had a strong effect on the response interval, with urgent contacts having a median response interval of 4:10 min compared to acute contacts with 2:01 min. This increase of 2:09 min was statistically significant (*p* < 0.0005). Increasing overall guideline adherence decreased EMD response interval (*p* < 0.0005) (Table 3).

**Table 3:** Overall guideline adherence and effect on EMD response interval^a^

	All calls (*N* = 299)		Ambulance dispatches (*N* = 279)				
					EMD response interval (min:sec)^a^		
	N	%	N	%	Median	IQR^b^	*p*-value
Overall guideline adherence							<0.0005
3	162	54	157	56	2:12	2:03	
2	101	34	92	33	3:19	3:56	
1	32	11	26	9	3:03	4:03	
0	4	1	4	1	4:28	10:36	0.225

^a^Emergency Medical Dispatch response interval: Time from 113 call is answered until an ambulance is dispatched

^b^ *IQR* Interquartile range

## Discussion

In this study we were able to measure actual use of the Index, and found a higher mean guideline adherence value compared to the previously published self-reported use of Index value [2]. Higher overall guideline adherence led to a statistically, significantly lower EMD response interval.

Considering that the guidelines are designed to function in cooperation with the operators’ skills and experience, it is difficult to designate a limit for acceptable use of the guidelines as a whole, and thus assess whether the mean guideline adherence of 80 % is acceptable or not. Start Page compliance, especially confirmation of patient location and consciousness, should be 100 % in an emergency medical call. The ideal compliance level with regards to the criteria cards will however be somewhat lower, due to the nature of the guidelines. The fact that the Index is still being used in a paper version in an otherwise electronic work environment can probably account for some of the situations where it is not used. Except occasional feedback from ambulance personnel on the scene, the EMCC operators never receive information on patient outcome. The relationship between guidelines and clinical outcome is known to be an important motivational factor for guideline adherence [5].

Although the 2009 study on impaired consciousness also found a discrepancy between self-reported use of Index (99 %) and measured use of Index (64 % confirmed consciousness) [7] the discrepancy is not only much wider, but also the opposite of our findings. This could indicate a positive development towards both higher actual use of Index and a more realistic view on own use.

The 2014 study comparing medical priority dispatch system (MPDS) and criteria-based dispatch (CBD) in cardiac arrest patients reported a guideline adherence of 97 % for successful clarifying level of consciousness [6]. This is higher than our finding that consciousness was clarified within 1 min in 83 % of the cases. One obvious explanation for this difference is their selection of highly acute contacts only, while our material includes all acute and urgent contacts. Furthermore, although not statistically significant, our study shows a wide variety between the EMCCs when it comes to percentage confirmed consciousness, ranging from 73 to 92 % for all acute and urgent cases in total. It has been shown that higher call processing rates increase dispatch performance. Kuisma et al. found in 2005 that operators processing >9 ventricular fibrillation cardiac arrest calls during the study period recognised cardiac arrest faster, dispatched first responding unit faster, gave cardiopulmonary rescusitation instructions and received bystander cooperation more often, and had a higher rate of patients discharged alive than dispatchers processing <4 VF calls during the study period [9].

Since the introduction of Index in Norway, it has been debated whether it saves time or adds time to the dispatch process. Of the six Index indicators investigated in our study, criteria compliance was the only one with a statistically significant effect on EMD response interval. The main effect on EMD response interval occurred between 1 and 2 unconfirmed criteria, with an increase of 1:31 (minutes:seconds) from 0 unconfirmed criteria to 2+ unconfirmed criteria. When looking at the combined outcome variable *overall guideline adherence*, we found a difference of 2:16 between the lowest and highest value group.

Failure to check vital information like consciousness or responsiveness obstruct detection of life-threatening situations like cardiac arrest or breathing difficulties where instructions from the operator might influence the outcome significantly. Berdowski et al. found in a study published in 2009 that failure to recognize cardiac arrest during the emergency call delayed both ambulance dispatch and arrival on the scene, and decreased 3-month survival from 14 to 5 % [10]. Using a similar protocol to our Start page, the Dutch dispatchers were supposed to clarify location, phone number, patient consciousness and breathing, and the study showed that failure to ask whether and how the patient was breathing was the primary reason for not recognizing the cardiac arrest. Previous versions of Index included breathing as a key question on the Start page; this was replaced by patient responsiveness in 2009 [1].

Low criteria compliance might result in a lower acuity than actually needed because important information is unrevealed, the patient might lose important time due to undertriage. A certain over triage is generally accepted in the prehospital emergency system, especially considering the difficulties of assessing a situation solely over the telephone, but undertriage may put the patient at unnecessary risk. An Italian study on dispatch errors focusing on patients initially coded as non-urgent and ending up as fatalities found that these calls were predominantly made by next of kin reporting not life-threatening symptoms, but they were also shorter and characterized by inadequate collection of vital information by the operators [11]. Andersen et al. studied a group of patients not identified as acute but who died the same day they were reported via a 112 call. The researchers found that although none of the deaths were considered definitively preventable, 12 % were considered potentially preventable if the call had been assessed as acute [12]. A Swedish study published in 2011 compared priority codes between the dispatch centre (CBD guidelines) and the ambulance on scene (Medical Emergency Triage and Treatment System—A, METTS-A) and found that although the Swedish index has a high sensitivity for identifying acute patients, 4.8 % of the patients were undertriaged [13]. A similar finding of 4.6 % undertriage was found in Switzerland in a study published in 2015 [14]. In Norway, experiences of general practitioners (GPs) participating in prehospital emergency situations show that 42 % of the patients are downgraded between initial dispatch centre triaging and on-the-scene triaging by a GP, while 11 % were upgraded, acute abdominal cases having the highest risk of being initially undertriaged [15].

### Strengths and limitations

Although only seven EMCCs took part in this study, they were strategically selected to be representative of the rest of the EMCCs and the results are therefore thought to be generalizable on a national level. This study thus provides valuable information on how and to what extent Index is actually used and how this affects the response interval.

The outcome variable *overall guideline adherence* derived from three of the six primary indicators, excluding telephone number confirmation, patient responsiveness and caller cooperation. Since the implementation of automatic number tracking of incoming calls, there seems to be a cultural acceptance at the EMCCs for only asking for the telephone number when the number is unregistered. Including this variable would have given a marked decrease in mean overall guideline adherence. The variable patient responsiveness provided little information beyond that provided by consciousness, and the numbers of uncooperative callers or language issues were very small. The cut-off value for the Start page indicators was chosen to be ‘within one minute’, as these are time-critical questions essential for further optimal handling of the situation. The cut-off for criteria compliance was set between one and two criteria not accounted for during the call, based on the effect on EMD response interval.

Our findings suggest higher guideline adherence compared to previous studies. Given the nature of CBD guidelines as an aid to the operator in the dispatch process, rather than the operator carrying out the dispatch process itself, one might question whether the present level of Index adherence is acceptable. However, further increased confirmation of consciousness and proper use of the criteria cards may reduce the risk of undertriage. Documenting overall guideline adherence is a further step towards exploring the validity of Index.

## Conclusion

The mean overall guideline adherence for acute and urgent emergency calls was 80 %. The results confirm previously self-reported use of Index findings. Low overall guideline adherence increases the EMD response interval and may increase the risk of undertriage.

## Additional files

Additional file 1: Index-113-registration, a call recordings registration form on adherence to different parts of index during the emergency call. (PDF 212 kb)
Additional file 2: Index-113calls work sheet, a data file supporting the conclusions of the article. (XLSX 64 kb)

## Abbreviations

AMIS: Acute Medical Information System
ANOVA: Analysis of variance
CBD: Criteria-based dispatch
CPR: Cardiopulmonary resuscitation
EMCC: Emergency Medical Communication Centre
EMD: Emergency medical dispatch
GP: General practitioner
IQR: Interquartile range
METTS-A: Medical Emergency Triage and Treatment System—A
MPDS: Medical Priority Dispatch System
SD: Standard deviation
The Index: The Norwegian Index for Medical Emergency Assistance
