# Supplementary material for: Dispatch guideline adherence and response interval—a study of emergency medical calls in Norway
Source: BMC Emerg Med. 2016 Oct 13;16:40. doi: 10.1186/s12873-016-0105-2 (PMC5064961; doi:10.1186/s12873-016-0105-2)
Supplement: Additional file 1: — Index-113-registration, a call recordings registration form on adherence to different parts of index during the emergency call. (PDF 212 kb) [file 12873_2016_105_MOESM1_ESM.pdf]

NORSK INDEKS FOR MEDISINSK NØDHJELP – BRUK AV INDEKS
LYDLOGGPROSJEKT

Sentral: ____________ AMIS: ____________ Dato: _____ Klokken: ____
Hovedproblem: ____
Innringer:_____________________________ (pasient, helsepersonell…)
Innringer samarbeider: Ja ◻ Nei ◻ ◻ Tredjehåndsinopplysninger

STARTKORT

HVOR - Hvor er pasienten/ulykken?

	Ja	Nei	Tid til avklart (</> 1 min)	Score
Adresse/stedsbeskrivelse verifisert?				
Tlf.nr verifisert?				

HVA - Hva er problemet?

____

____

____

VÅKEN - Er pas. våken og i stand til å snakke?

Spm. stilt/registrert	Ja	Åpenb/unødv	Nei	Tid til avklart (1 min)	Score
Er pasienten våken?					
Er pasienten i stand til å snakke?					
Reagerer på tilrop og risting?					
Alder?					
Kjønn?					
Navn?					
Full ID?					
Fastlege?					

**STARTKORT**		**RESPONS**	
**TILSTAND/HASTEGRAD**		**RÅD/VEILEDNING/INSTRUKSJON**	
**TOTALT:**			

TILSTAND / HASTEGRAD

Satt kriterie __________ Satt hastegrad _______________ Enig? _________________________

A	Ja	Åpenb/unødv	Nei	H	Ja	Åpenb/unødv	Nei	V	Ja	Åpenb/unødv	Nei
01				01				01			
02				02				02			
03				03				03			
04				04				04			
05				05				05			
06				06				06			
07				07				07			
08				08				08			
09				09				09			
10				10				10			
11				11				11			
12				12				12			
13				13				13			
14				14				14			

Vurdert kriterie _____________________ Vurdert hastegrad _________________________

RESPONS

	Ja	Åpenb./unødv .	Nei	Score	Kommentar
Tiltak ihht hastegrad/kriterie?					
Legevaktlege alarmert?					

RÅD/VEILEDNING/INSTRUKSJON

	Ja	Åpenb./unødv.	Nei	Score	Kommentar
Riktig å tilby RVI?					
Mulig å tilby RVI?					
RVI tilbudt?					
RVI korrekt?					
Instruksjon akseptert?					
